# A Twisted Cecum: Herniation and Volvulus of the Cecum Through the Epiploic Foramen

**DOI:** 10.7759/cureus.27754

**Published:** 2022-08-07

**Authors:** Deepa Chandhrasekhar, Ariel Droger, Margaux Baatz, Timothee Chapuis, Debora J Fox-McClary

**Affiliations:** 1 School of Osteopathic Medicine, Midwestern University Arizona College of Osteopathic Medicine, Glendale, USA; 2 General Surgery, Abrazo Community Health Network, Glendale, USA; 3 Colon and Rectal Surgery, Abrazo Community Health Network, Glendale, USA

**Keywords:** blandin's hernia, lesser sac hernia, foramen of winslow herniation, ileocecal herniation, cecal volvulus, internal hernia

## Abstract

Herniation of the cecum, terminal ileum, and ascending colon through the epiploic foramen is an uncommon presentation of an internal hernia. An 82-year-old female presented with a small bowel obstruction; Computed Tomography (CT) imaging showed a herniation of the terminal ileum, cecum, and ascending colon through the foramen of Winslow into the lesser sac, with cecal volvulus. Prompt surgical treatment included laparotomy and reduction of the hernia, followed by an extended right hemicolectomy with primary anastomosis and functional closure of the epiploic foramen. This report reviews the natural history and management of this rare pathology.

## Introduction

An internal hernia is a projection of abdominal viscera through a peritoneal or mesenteric defect or window in the abdominal or pelvic cavity [1]. Internal hernias of the bowel through the epiploic foramen or foramen of Winslow, also known by the eponym Blandin’s hernia, account for approximately 8% of internal hernias [2-4]. The estimated overall incidence of cecal herniation through the foramen of Winslow is reported to be 0.02% [5]. A current PubMed search of case reports using keywords “cecal volvulus” and “foramen of Winslow” returned nine published case reports within the last 10 years.

Owing to the paucity of experience, no standard surgical approach exists for the correction of internal hernias through the epiploic foramen. Both open and laparoscopic methods of reduction are described, with the vast majority of cases performed using laparotomy. Less than 10% of internal hernias through the epiploic foramen are diagnosed preoperatively [4]. Often, definitive care is delayed because the full extent of the herniation and any potential strangulation of the bowel is not recognized until the patient is in the operating room (OR); consequently, these cases have a mortality of up to 50% [4]. With prompt CT and early surgical intervention, mortality can be reduced to 5% [4].

Symptom onset can be acute or subacute, typically accompanied by signs and symptoms of intestinal obstruction [4]. Diagnosis is made based on physical exam and imaging, and definitive care requires surgery [4]. Here, we present a case with subacute onset with progression to small bowel obstruction due to an internal herniation of the distal small bowel, cecum, ascending colon, and hepatic flexure through the epiploic foramen into the lesser sac with associated cecal volvulus. The patient was managed with urgent surgical intervention. 

## Case presentation

The patient was an 82-year-old previously healthy female with a history of multiple deep vein thromboses, factor V Leiden deficiency on warfarin, and mild dementia. She denied chronic constipation or prior abdominal surgery. Two weeks before the presentation, the patient stated she accidentally fell hard onto her back but did not seek medical care. Shortly after this fall, she developed mild abdominal pain, abdominal distention, and constipation. She attempted to treat her symptoms using over-the-counter stool softeners and, in fact, had a bowel movement one day before the presentation. In the 24 hours before the presentation, she developed increasing abdominal distention with new onset of nausea and vomiting, leading to her index urgent care visit.

Initial vitals were within normal limits with a body mass index (BMI) of 28.7. On physical exam, the patient was alert and oriented and in no acute distress. The abdomen was soft, mildly distended, and mildly tender to palpation in both upper quadrants, with the absence of peritoneal irritation. Initial labs were notable for borderline sodium of 135, creatinine 1.7, blood urea nitrogen (BUN) 26, lipase 180 U/L, and international normalized ratio (INR) 3.7.

**Figure 1:**
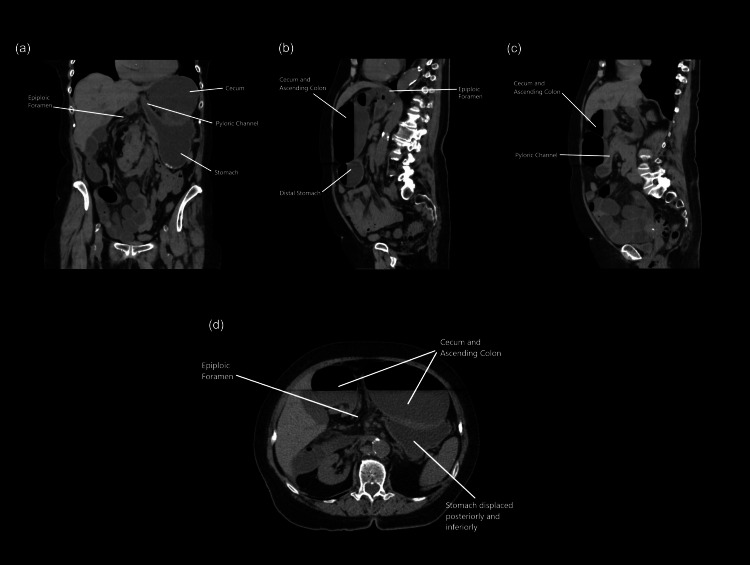
CT abdomen and pelvis without contrast (a-d). The cecum is shown herniating through the epiploic foramen of Winslow and posterior to the pyloric channel.

**Figure 2:**
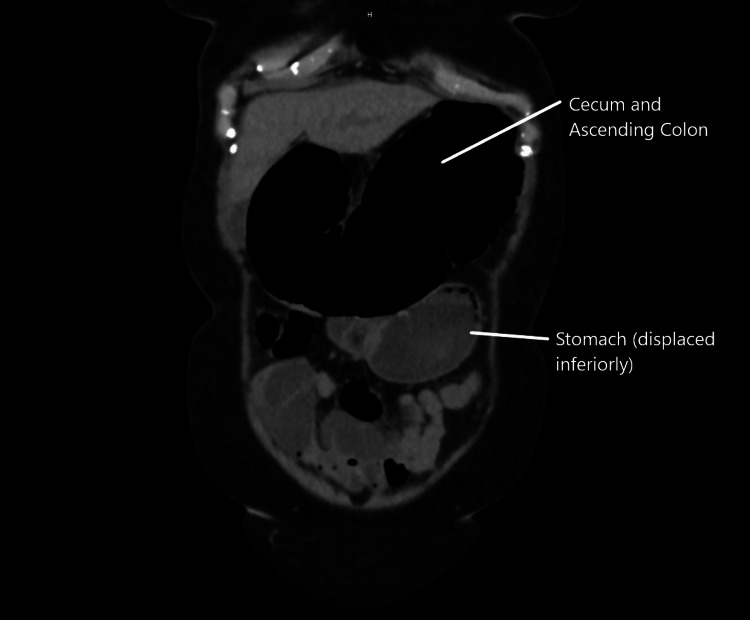
CT abdomen and pelvis without contrast. Distended cecum and ascending colon shown herniating through the epiploic foramen of Winslow (not shown). The cecum and ascending colon inferiorly displaced the stomach.

**Figure 3:**
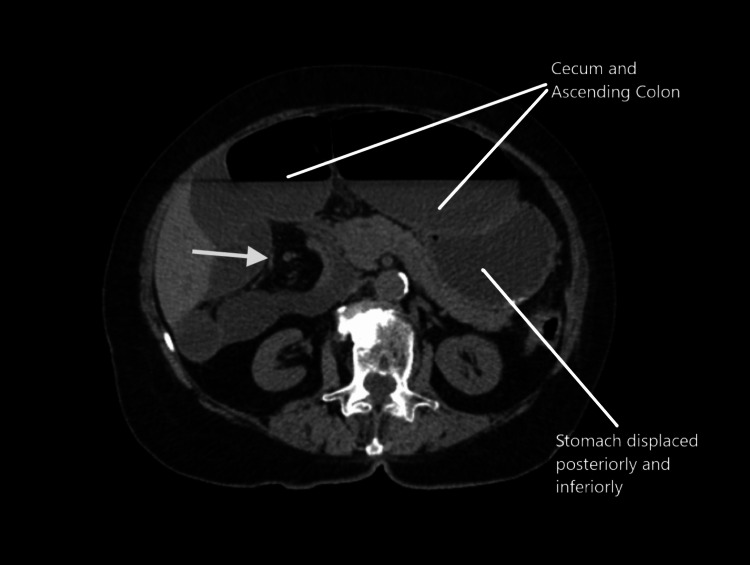
CT abdomen and pelvis without contrast. Whirl sign displaying cecal volvulus (arrowhead).

CT imaging demonstrated rotation and herniation of the cecum, ileum, and a portion of the ascending colon through the foramen of Winslow into the lesser sac without secondary signs of bowel ischemia. The cecum and appendix were positioned within the lesser sac, posterior to the stomach, and near the gastroesophageal junction. The hernia site through the foramen created a transition point for a coexistent small bowel obstruction. A whirl sign was noted by the radiologist, suggesting a cecal volvulus. Figures 1-3 below show the CT imaging, and Video 1 shows a coronal CT of the abdomen and pelvis.

**Video 1:** CT abdomen and pelvis without contrast, coronal view. The cecum can be seen herniating into the foramen of Winslow with a whirl sign indicating cecal volvulus.

A nasogastric (NG) tube was placed to low intermittent suction with an immediate return of 650 ccs of bilious enteric contents. The patient was otherwise stabilized and urgently transferred to our facility.

Upon arrival at our center, the patient appeared comfortable and was hemodynamically stable. She stated that the pain and distention of her abdomen had mostly resolved, and she was passing flatus. On physical exam, her abdomen was distended and tympanitic but minimally tender, lacking peritoneal signs. The patient received fluid resuscitation and was decompressed with an NG. Purgative bowel preparation was not appropriate in this patient; however, an oral antibiotic preparation of neomycin and metronidazole was initiated for colon decontamination in anticipation of resection. The use of prothrombin complex concentrate (Kcentra) was considered to correct her anticoagulation; however, since the patient was stable and minimally symptomatic, we opted for a more cost-effective method of reversal using 10 mg of intravenous (IV) vitamin K and two units of fresh frozen plasma (FFP). Within hours of arrival, an INR of 1.42 was achieved, and the patient was taken to the OR. 

Laparotomy was initiated with a limited periumbilical midline incision that later required extension to a full midline incision. Blood-tinged ascites were aspirated upon entry into the abdomen. The terminal ileum, cecum, ascending colon, and hepatic flexure were herniated through the epiploic foramen into the lesser sac. A massively dilated colon was visible through a thin gastrohepatic ligament above the lesser curvature of the stomach. This portion of the bowel appeared viable but was slightly dusky in color. 

Reducing the hernia was challenging. Several techniques were utilized in attempting to reduce the hernia. 1) Direct manual extraction of the terminal ileum and cecum was attempted, placing gentle pressure on the bowel in the peritoneal cavity proper just before it entered the epiploic foramen, trying to extract the bowel from the lesser sac. This was unsuccessful as the bowel was firmly fixed within the lesser sac. 2) The duodenum was fully kocherized to enlarge the epiploic foramen, a technique that proved to be technically difficult given the severe distortion of anatomy in this area. This was unsuccessful as the herniated bowel remained fixed in the lesser sac and could not be manually extracted. 3) The greater omentum was mobilized off of the transverse colon, and the lesser sac was entered, after which manual pressure was applied from within the lesser sac to try to return the dilated bowel to the peritoneal cavity properly. The herniated bowel remained firmly fixed within the lesser sac. 4) A combination of applying pressure from within the lesser sac and simultaneously attempting direct manual extraction from within the peritoneal cavity proper was attempted but was unsuccessful as the bowel would not reduce. 5) A purse string suture was placed in the anterior aspect of the dilated cecum, after which an intentional cecal enterotomy was created through which cecal contents were evacuated with pool tip suction. The suture was tied down after evacuation to avoid peritoneal contamination. Finally, the bowel within the lesser sac was reduced and returned to the peritoneal cavity properly.

The nurse anesthetist was cautioned that upon reduction of the hernia the patient might experience reperfusion-related hemodynamic instability. Shortly after reduction, the patient developed transient hypotension and was quickly stabilized with fluid resuscitation and vasopressor support. 

After reduction, the cecum, ascending colon, and proximal transverse colon appeared elongated, floppy, and hypermobile. The bowel remained slightly dusky in color, and the cecum showed patchy ischemic changes. Due to possible ischemia, the presence of the therapeutic cecal enterotomy, and concern for recurrence of the hernia or volvulus, an extended right hemicolectomy was performed with a primary anastomosis.

A portion of omentum originating from the right aspect of the greater curvature of the stomach was brought into the lesser sac and through the epiploic foramen from medial to lateral in order to deter future herniation through this site. This was not sutured in place. The peritoneal cavity was irrigated, hemostasis was confirmed, and the abdomen was closed.

The patient was taken directly from the OR to the intensive care unit, hemodynamically stable, and on minimal vasopressor support. The patient was extubated shortly, and pressors were weaned. Given her hypercoagulability disorder, the surgical manipulation around the portal triad, and other comorbidities, anticoagulation with subcutaneous heparin was started to deter the development of portal vein thrombosis or another systemic thrombosis. A clear liquid diet was started the morning following surgery and advanced to a regular diet uneventfully. IV fluids were discontinued once the patient tolerated a diet.

On postoperative day four, the patient experienced 18 minutes of ECG confirmed supraventricular tachycardia (SVT) at 210 beats per minute, during which time her blood pressure remained stable. The rhythm spontaneously reverted to normal sinus. Troponins were slightly elevated at 0.08 ng/L (normal 0 to 0.02 ng/L), but a stat follow-up echocardiogram was normal. Beta-blocker therapy was initiated and SVT did not reoccur. The patient was discharged home on postoperative day seven. Warfarin was restarted before discharge with plans for an enoxaparin bridge.

## Discussion

Internal herniation of the terminal ileum, cecum, and ascending colon through the epiploic foramen into the lesser sac is an uncommon problem; as a result, the etiology and natural history of this condition remains uncertain. Three main mechanisms have been proposed for the pathogenesis: hypermobile viscera, intra-abdominal pressure changes, and abnormal enlargement of the foramen of Winslow [3]. The presence of an abnormally long bowel, a “wandering cecum”, failure of the right colon to become retroperitoneal, and defects of the gastrohepatic ligaments have been reported as predisposing factors [3, 6-7]. The literature on cecal volvulus suggests that hypermobility of the cecum and ascending colon may predispose the patient to developing volvulus, occurring either as a result of congenitally absent retroperitoneal attachments or due to acquired lengthening of these attachments [8]. Risk factors for acquired hypermobility include chronic constipation, neuropsychological impairment, and the presence of multiple comorbidities [8], This patient was relatively healthy, denied constipation until two weeks before presentation, and exhibited very mild dementia, with normal functioning prior to hospital admission. Intraoperatively this patient lacked the usual and typical retroperitoneal attachments of these structures, allowing for hypermobility. Her symptoms started immediately after her fall, suggesting trauma may have acutely contributed to her presentation, perhaps through the hypothesized intra-abdominal pressure changes.

Internal hernias through the epiploic foramen have been classified based on the organ(s) involved in the hernia: type I (small bowel, 65% of cases), type II (terminal ileum, cecum, and ascending colon, 25% of cases), type III (transverse colon, 7% of cases), or type IV (gallbladder or any other intraperitoneal structure such as the greater omentum, 3% of cases) [3]. Using this classification system, this patient had a type II epiploic foramen hernia. 

Operative management is the definitive treatment, and an open approach is most commonly used. Operative correction may be urgent or emergent depending on the patient's status, with emergent correction required if there is clinical evidence of bowel ischemia. In this case, gentle traction was not useful in reducing the hernia. Open or needle decompression of the bowel can aid in hernia reduction, especially if the bowel is dilated [3]. The epiploic foramen can be expanded to assist in the reduction in cases of massive colonic dilatation or edema through a wide Kocher maneuver [3]. In our case, gentle traction was not useful in reducing the hernia. Both widenings of the epiploic foramen, and open cecal decompression contributed to the successful reduction of the hernia, though the distortion of the right upper quadrant anatomy made duodenal kocherization technically challenging.

It is unclear if the closure of the epiploic foramen is necessary to prevent hernia recurrence. In the limited published literature, rates of hernia recurrence within 21 months of the operation were similar whether or not the epiploic foramen was closed [3]. Suture closure of the epiploic foramen has been described, though such closure must be done cautiously to prevent iatrogenic injury to the surrounding portal structures [3]. If the cecum and ascending colon are viable, fixation within the right paracolic gutter via cecopexy has been described [3]. Garg et al. simultaneously closed the foramen with an omental patch and secured the colon with cecopexy [9]. In multiple case reports, no prophylactic measures were described to occlude the foramen. In this case, omentum was purposely brought through the foramen to occupy the opening, a novel method not previously reported, creating a purposeful type IV hernia while avoiding the dangers of suture closure.

## Conclusions

Internal herniation of the small bowel and right colon through the epiploic foramen is an uncommon cause of small bowel obstruction. Such hernias carry an associated risk of bowel ischemia or strangulation; thus, presentation may be acute or subacute in onset. Imaging defines the anatomy and confirms the presence of internal herniation. Surgery may be urgent or emergent, depending on the clinical presentation, and is required for definitive correction of the disorder.

Patients should be medically optimized, and surgical correction completed expeditiously to optimize patient outcomes. As illustrated in this case, reducing the herniated bowel from the lesser sac through the epiploic foramen can be challenging. The bowel, once reduced from the hernia, lacks the usual and customary congenital peritoneal attachments and is highly mobile; thus, most surgeons provide fixation through cecopexy or perform resection in an attempt to deter recurrence of the internal hernia. The necessity of closing the epiploic foramen is unclear, and suturing in the region of the foramen may further add to the previously discussed risks. Case reports on internal herniation through the epiploic foramen remain infrequent, and no treatment or prevention consensus exists at this time. This case presentation contributes to the current literature by illustrating a rare diagnosis, reviewing surgical strategies to aid the surgeon, and providing a unique method to “closing” the epiploic foramen.
